# The stem cell inhibitor salinomycin decreases colony formation potential and tumor‐initiating population in docetaxel‐sensitive and docetaxel‐resistant prostate cancer cells

**DOI:** 10.1002/pros.23940

**Published:** 2019-12-13

**Authors:** Martina Gruber, Florian Handle, Zoran Culig

**Affiliations:** ^1^ Department of Urology, Division of Experimental Urology Medical University of Innsbruck Innsbruck Austria; ^2^ Department of Cellular and Molecular Medicine Molecular Endocrinology Laboratory, KU Leuven Leuven Belgium

**Keywords:** automated analysis, colony formation, colony types, inhibition of holoclones, prostate cancer stem cells

## Abstract

**Background:**

Prostate cancer (PCa) is one of the most frequently diagnosed tumors in men. In general, therapies for localized PCa are curative. However, treatment of advanced PCa is considered palliative since development of therapy resistance occurs rapidly. It has been shown that tumor‐initiating cells are likely involved in therapy resistance. They are not eliminated by conventional therapies and thereby lead to tumor progression and relapse. The aim of this study was to evaluate the effects of the known stem cell inhibitor salinomycin on this critical subpopulation of cells.

**Methods:**

Expression of the cell surface markers CD24 and CD44 was assessed by immunofluorescence and fluorescence‐activated cell sorting. Colony formation efficiency and classification of colony types with varying tumor‐initiating potential (holoclones, meroclones, and paraclones) were analyzed in an automated way by the newly developed CATCH‐colonies software in the absence or presence of salinomycin.

**Results:**

Automated high‐resolution colony formation analysis consistently identified the various colony types in a broad range of PCa cell lines. Serial clonogenic assays confirmed that holoclones show the highest colony formation potential and maintain their tumor‐initiating capacity over multiple rounds. Furthermore, holoclones showed high expression of CD44, while CD24 was not expressed in these clones, thus representing the well‐described tumor‐initiating CD24^−^/CD44^high^ population. Salinomycin decreased the CD24^−^/CD44^high^ population in both docetaxel‐sensitive PC3 and docetaxel‐resistant (DR) PC3‐DR. Moreover, treatment of PC3, DU145, PC3‐DR, and DU145‐DR with salinomycin led to a significant reduction in the colony formation potential by targeting the colonies with high tumor‐initiating potential.

**Conclusions:**

Taken together, we demonstrated that salinomycin specifically targets the tumor‐initiating cell population in docetaxel‐sensitive and docetaxel‐resistant PCa cells and may represent a potential therapeutic approach for the treatment of advanced PCa.

## 1 INTRODUCTION

Prostate cancer (PCa) is one of the most frequently diagnosed malignant tumors in men. Although therapy of localized PCa is curative, treatment of advanced PCa is considered palliative. Therapies for patients with biochemical and clinical recurrence predominantly target the androgen receptor (AR). However, these treatments inevitably lead to the development of castration‐resistant PCa within a few years. A possible explanation for development of resistance to androgen‐directed therapies is the existence of cancer stem cells, which were described in detail by Maitland et al.^1^ They postulate that there is a hierarchy within tumors and that the bulk population of tumor cells is derived from tumor‐initiating cells, which represent a small self‐renewing subpopulation.^2^ They may belong to the basal compartment of the prostate,^1^ show no AR expression and thereby are not targeted by conventional therapies.^3^ The issue of the origin of PCa is still open for discussion. Other researchers have described the role of prostate luminal progenitor cells in tumorigenesis.^4^ However, there is still much discussion on how these aggressive cells can be identified and consequently targeted. Barrandon and Green^5^ have described more than 30 years ago that single cells can form three morphologically different colony types with varying tumor‐initiating potential: paraclones (low), meroclones (intermediate) and holoclones (high tumor‐initiating potential). These distinct clonal colony types can be identified based on their morphology and marker expression^6^ are typically described. Several research groups have demonstrated with serial transplantation assays in vitro and in vivo in mice that holoclones contain the cells capable of indefinite self‐renewal.^7^, ^8^ Similar observations were made by Shimada et al^9^ who demonstrated the appearance of syndecan‐1, which is important for stabilization of stem cells, in holoclones. Moreover, Zhang and Waxman^10^ as well as Beaver et al^11^ showed that holoclones display the highest tumorigenic potential when inoculated into mice, in contrast to paraclones.

The aim of this study was to analyze the effects of the stem cell inhibitor salinomycin, which has been described by Dewangan et al.^12^ In particular, we determined the impact of salinomycin on the tumor‐initiating CD24^−^/CD44^high^ population.^13^ Furthermore, we evaluated the effects of salinomycin on colony formation efficiency and distribution of colony types in docetaxel‐sensitive and docetaxel‐resistant cells using automated high‐resolution colony formation analysis. Salinomycin has been used in docetaxel‐resistant cells because those cells are known to express stem‐like properties^14^, ^15^ and the combination of salinomycin and docetaxel was earlier proposed to be a promising strategy to target both gastric cancer cells and cancer stem cells.^16^

## 2 MATERIALS AND METHODS

### 2.1 Cell culture

Human cell lines PC3, DU145, and LNCaP were obtained from American Type Culture Collection (ATCC, Rockville, MD). LAPC4 were a kind gift from Prof. A. Cato (Institute of Toxicology and Genetics, Karlsruher Institut für Technologie, Germany). Docetaxel‐resistant PC3‐DR and DU145‐DR were previously established by Puhr et al.^14^ All cells were cultured in Roswell Park Memorial Institute 1640 (RPMI‐1640) (PAN Biotech, Aidenbach, Germany) containing 10% (v/v) fetal calf serum (PAN Biotech), 1% (v/v) penicillin/streptomycin and 1% (v/v) GlutaMAX (both from Lonza, Vienna, Austria). LNCaP were supplemented with 1% (v/v) 4‐(2‐hydroxyethyl)‐1‐piperazineethanesulfonic acid (HEPES) (Sigma, Vienna), 1% (v/v) d‐glucose (Sigma), 1% (v/v) Na‐pyruvate (Lonza) and LAPC4 with 100 nmol/L dihydrotestosterone (Sigma). PC3‐DR and DU145‐DR were cultured in the presence of 12.5 nmol/L docetaxel (Sigma). The authenticity of all cell lines was validated via short tandem repeat profiling.

### 2.2 High‐resolution colony formation analysis

Limiting cell numbers (1000 cells for PC3, DU145, and PC3‐DR, 2000 cells for DU145‐DR) were seeded in T75 cell culture flasks and incubated for 10 to 14 days. The exact number of viable cells was determined using CASY cell counter system (Schärfe System, Reutlingen, Germany). After the incubation time cells were fixed with 100% ice‐cold methanol for 5 minutes and stained with crystal violet (0.5% in phosphate‐buffered saline [PBS] containing 20% methanol; Sigma) for 5 minutes. The flasks were filled with pure white starch powder to increase the contrast and subsequently scanned on a flat‐bed scanner (CanoScan Mark II; Canon Austria GmbH, Vienna) with a resolution of 4800 dpi. Colony formation efficiency and distribution of colony types were analyzed using the CATCH‐colonies software (https://catch-colonies.net/). Serial clonogenic assays were performed in 96‐well plates by adjusting the seeding density to ensure the formation of single colonies in individual wells. Colony numbers and types were assessed manually under a microscope before trypsinization and reseeding of cells.

### 2.3 Immunofluorescence staining

Cells were seeded on glass coverslips and incubated until single colonies have formed. Following antibodies and isotype controls were used: fluorescein isothiocyanate (FITC) Mouse Anti‐Human CD24 (1:5; Becton Dickinson, Heidelberg, Germany), PerCP‐Cy5.5 Mouse Anti‐Human CD44 (1:50; Becton Dickinson), FITC mouse IgG1 κ isotype control (Becton Dickinson) and PerCP‐Cy5.5 mouse IgG2b isotype control (Becton Dickinson). Antibody incubation was performed in PBS containing 1% bovine serum albumin for 1 hour at 4°C. Colonies were visualized using fluorescent microscopy on a Zeiss Axio Imager microscope.

### 2.4 Flow cytometry

Cells were seeded in multiwell plates and treated with the indicated concentrations of salinomycin (Selleck Chemicals, Munich, Germany) for 72 hours. Cells were harvested, washed in PBS and resuspended in PBS containing 1% bovine serum albumin. The same antibodies, concentrations and incubation times as for immunofluorescence staining were used. After several washing steps, cells were analyzed by a BD FACS Calibur Flow Cytometer (Becton Dickinson).

### 2.5 RNA isolation and quantitative real‐time polymerase chain reaction

Total RNA was isolated using the EXTRACTME TOTAL RNA KIT (Lab Consulting, Vienna) according to the manufacturer's instructions. cDNA synthesis was performed with the iScript Select cDNA Synthesis Kit (Bio‐Rad, Hercules, CA). A Luna Script RT Super Mix Kit (New England Biolabs. Ipswich, MA) was used for real‐time polymerase chain reaction (PCR). As endogenous controls HPRT1, TATA‐Box binding protein and HMBS were used. For Nanog, ALDH1A3 and OCT4 (POU5F1) the following TaqMan gene expression assays from Thermo Fisher Scientific were used: Hs04399610_g1, Hs00167476_m1, and Hs04260367_gH.

### 2.6 Statistical analysis

Statistical analysis was performed using GraphPad Prism 5 (GraphPad Software Inc., San Diego, CA). Differences between control and treatment groups were analyzed using the Student *t* test. *P* < .05 was considered statistically significant and encoded as follows: *P < .05; **P < .01. All experiments have been performed in at least three biological replicates.

## 3 RESULTS

### 3.1 Identification, characterization, and automated analysis of colony types in PCa cells

**Figure 1:**
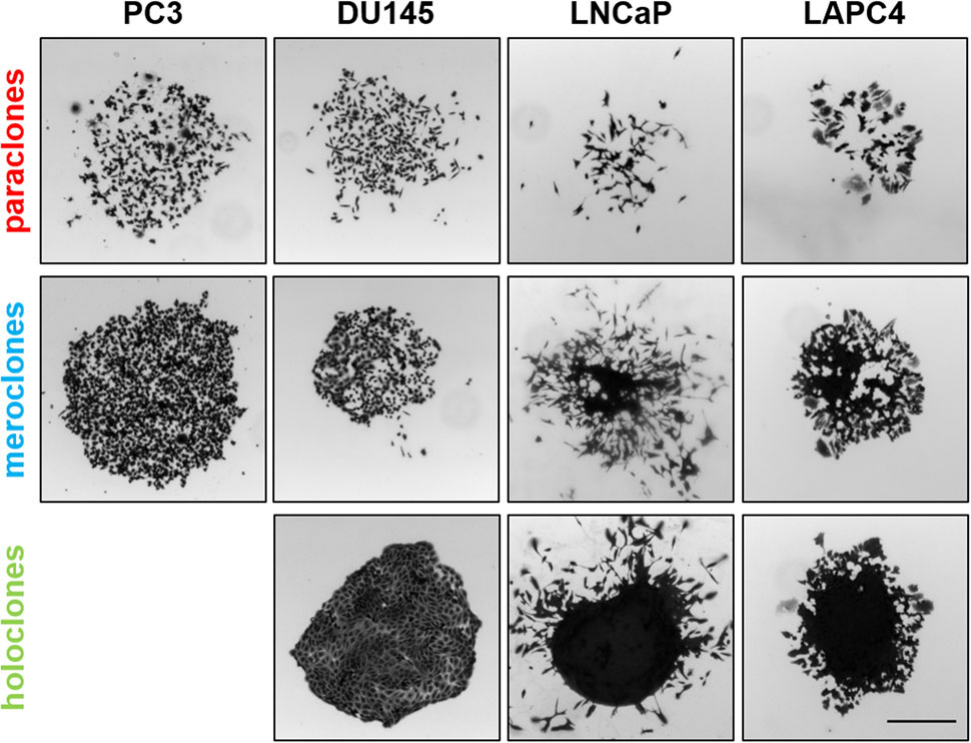
Identification of colony types in several prostate cancer (PCa) cell lines. Representative light microscopy images of crystal violet stained colony types (paraclones, meroclones, and holoclones) in various PCa cell lines (PC3, DU145, LNCaP, and LAPC). Scale bar = 500 µm [Color figure can be viewed at wileyonlinelibrary.com]

To investigate the three previously described colony types,^5^ several PCa cell lines were seeded at low density and grown for 10 to 14 days. In general, the three colony types were found in all cell lines tested with the exception of PC3. Figure 1 shows representative images of the colony types with contrasting morphologies. Paraclones have a quite irregular structure with loosely packed cells that show the highest grade of differentiation. Holoclones, on the other hand, are tightly packed and very compact. Meroclones are semisolid, but they do not have the same dense structure as holoclones.

**Figure 2:**
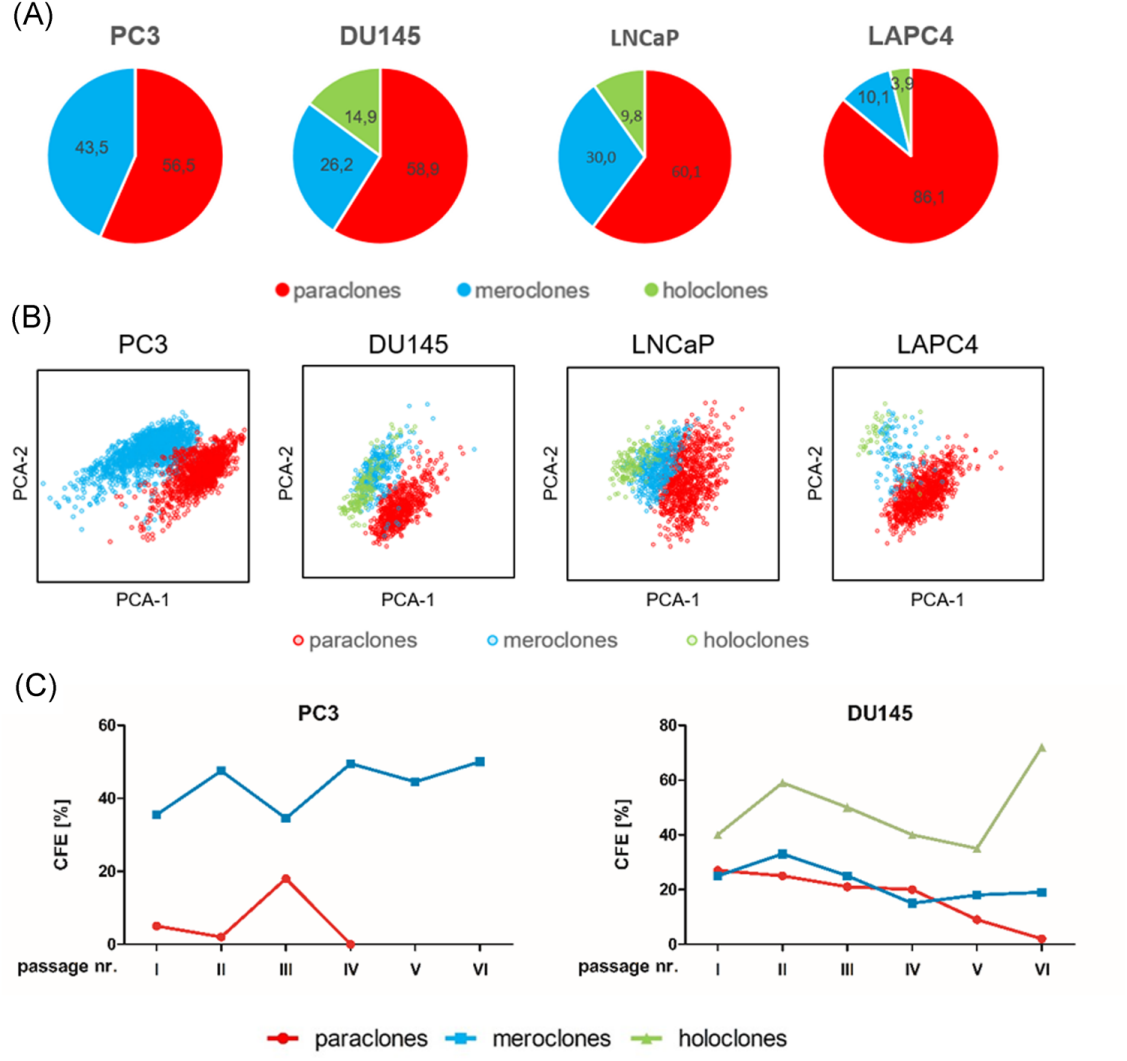
Automated classification of colony types by the software CATCH‐colonies. A, Quantification of the three colony types (red, paraclones; blue, meroclones; green, holoclones) in PC3, DU145, LNCaP, and LAPC4. B, Clustering of different colony types by principal component analysis (PCA) after analysis by the CATCH‐colonies software. C, Serial clonogenic assays of colony types in PC3 and DU145 (n = 1, the experiment was performed to ensure that identification of the colony types is in accordance with the work of others^7^) [Color figure can be viewed at wileyonlinelibrary.com]

In this study, the classification of colony types was performed in an automated way using the software CATCH‐colonies. In all cell lines, paraclones form the largest segment (from 56% in PC3 to 86% in LAPC4, Figure 2A), followed by meroclones (from 10% in LAPC4 to 43% in PC3). Holoclones form the smallest part (from 0% in PC3 to 15% in DU145), which is in agreement with other publications.^7^, ^17^ Figure 2B shows the clustering of the different colony types in various PCa cell lines by principal component analysis (PCA). To verify the characteristics of the identified colony types, we analyzed the mRNA expression levels of several stem cell markers (Nanog, ALDH1A3, and OCT4) in DU145 cells. The majority of stem cell markers were significantly upregulated in holoclones compared to paraclones (Figure S1A). Subsequently, we performed serial clonogenic assays to further confirm the correct annotation of the individual colony types. Paraclones showed the lowest colony formation efficiency in PC3 and DU145 cells and lost their proliferative potential after a few passages (Figure 2C). In contrast, meroclones and holoclones could be passaged over numerous rounds, which confirms their self‐renewing potential. Of note, in PC3 cells did not give rise to classical holoclones with a dense inner core; however, meroclones in PC3 maintained a very high colony formation efficiency similar to holoclones in DU145 cells (Figure 2C). Subcultivation of single colonies predominantly led to the formation of daughter‐colonies of their respective type (Figure S1B). Most importantly, only holoclones were able to give rise to all three types of colonies which has also been observed by others.^7^

### 3.2 Salinomycin treatment reduces tumor‐initiating CD24^−^/CD44^high^ population

**Figure 3:**
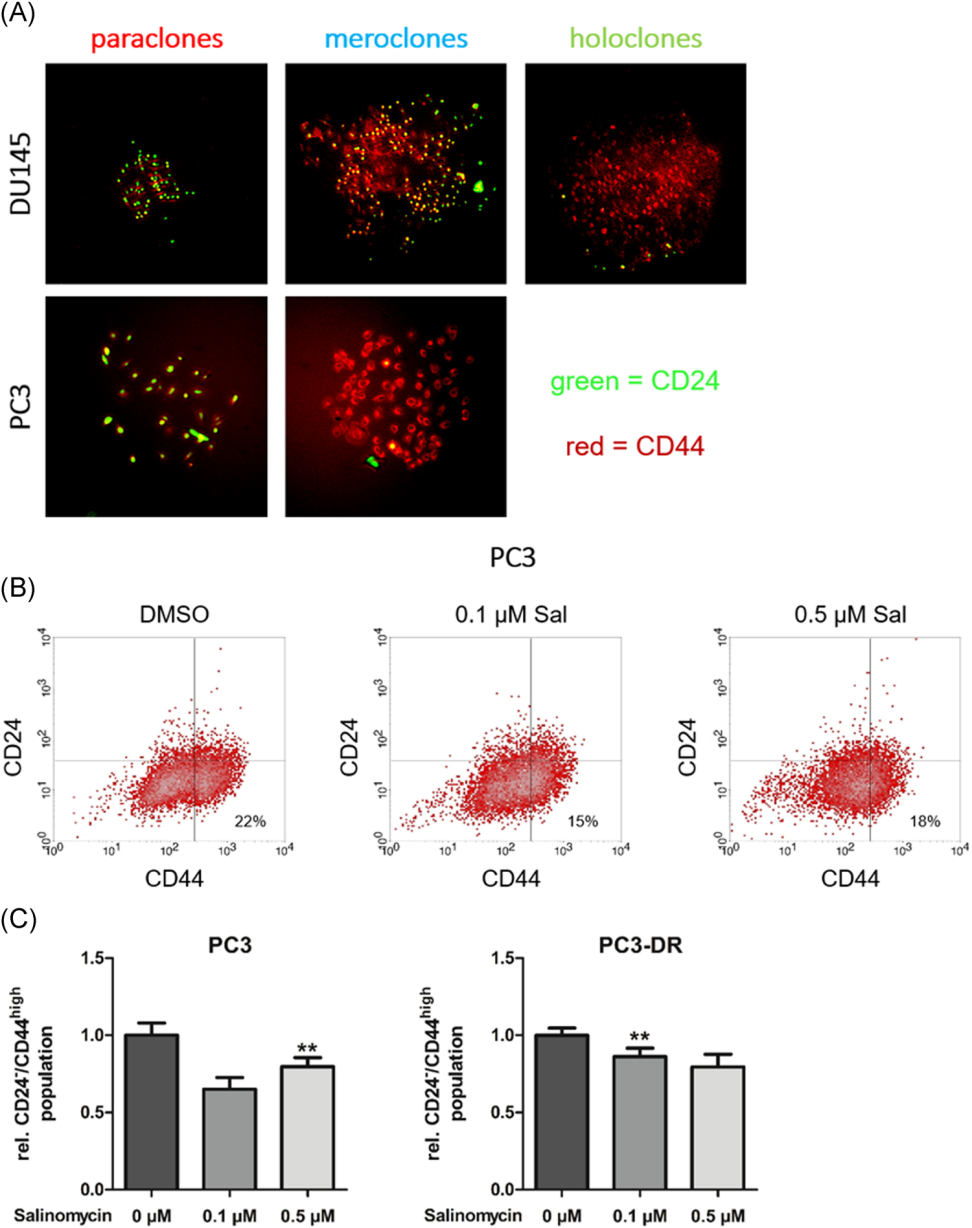
Salinomycin treatment decreases the tumor‐initiating CD24^−^/CD44^high^ population. A, Immunofluorescence staining for CD24 (green) and CD44 (red) in colony types of DU145 and PC3. B, Effect of salinomycin on tumor‐initiating CD24^−^/CD44^high^ population of PC3 and PC3‐DR cells was measured by fluorescence‐activated cell sorting (FACS). C, Quantification of CD24^−^/CD44^high^ population. Data represent mean ± SEM from three independent experiments (**P < .01; *t* test) [Color figure can be viewed at wileyonlinelibrary.com]

To validate the connection between identified colony types and the well‐described tumor‐initiating CD24^−^/CD44^high^ cell population we performed immunofluorescence staining and fluorescence‐activated cell sorting (FACS). Immunofluorescence staining revealed that holoclones in DU145 and meroclones in PC3 contain CD44‐positive cells that do not express CD24 (Figure 3A). Paraclones displayed high CD24 expression, but low CD44 expression. Meroclones in DU145 showed an intermediate phenotype with a moderate double‐positive expression of both cell surface markers. In the next step, the effects of salinomycin on this population were determined by performing FACS analysis. Indeed, salinomycin significantly decreased the CD24^−^/CD44^high^ population in both PC3 and PC3‐DR cells in bulk experiments (Figures 3B and 3C).

### 3.3 Salinomycin suppresses the formation of colonies with high tumor‐initiating potential

**Figure 4:**
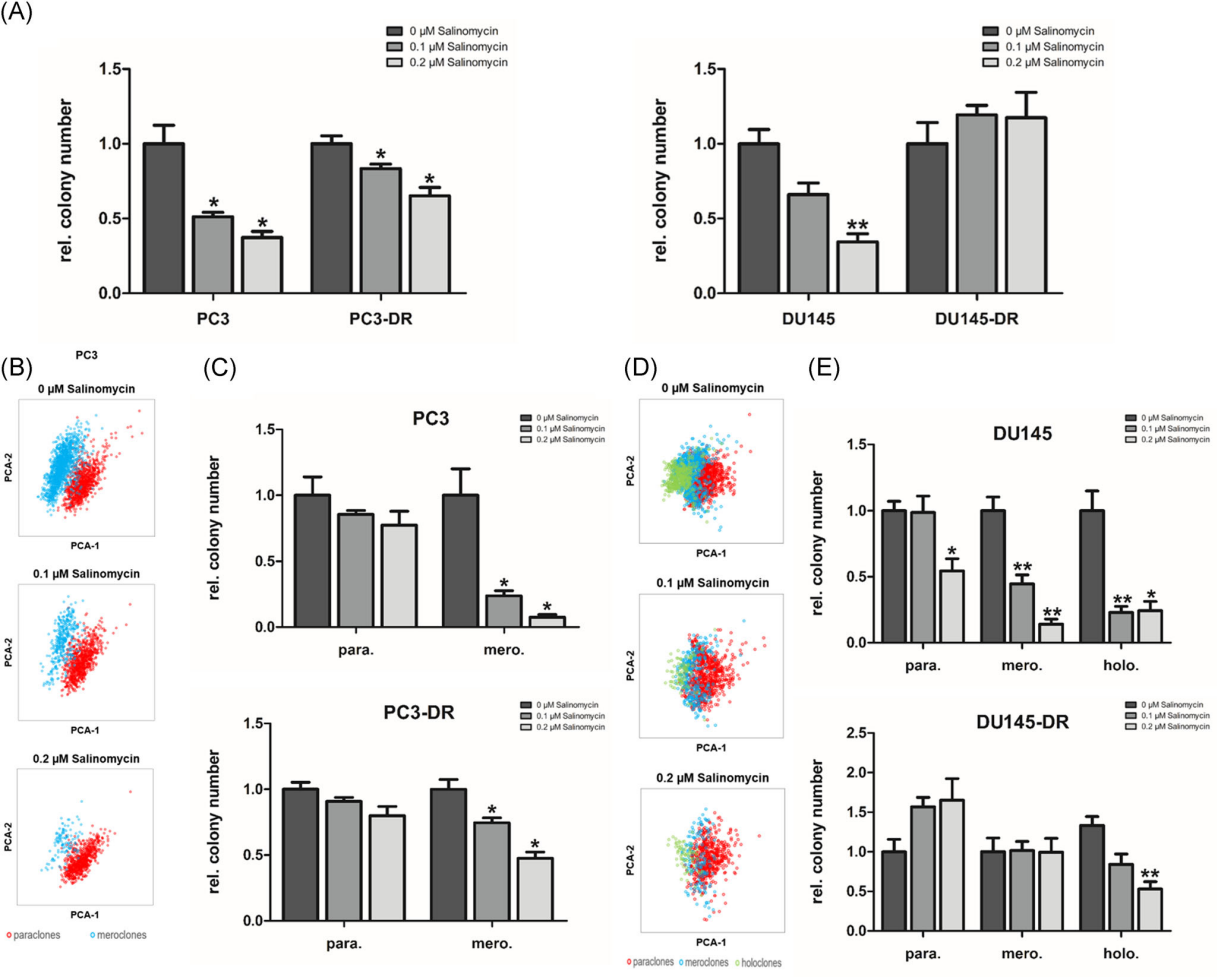
Salinomycin decreases colony formation efficiency by targeting tumor‐initiating clones. A, Colony formation efficiency of PC3, PC3‐DR, DU145, and DU145‐DR treated with the indicated concentrations of salinomycin was assessed by determining colony numbers after 10 days. Quantification was performed by the CATCH‐colonies software. Data represent mean ± SEM (*P < .05; **P < .01; *t* test). B, Clustering of colony types in PC3 treated with the indicated concentrations of salinomycin (paraclones, red; meroclones, blue). C, Relative number of each colony type in PC3 and PC3‐DR upon salinomycin treatment was analyzed by the software CATCH‐colonies. Values indicated are mean ± SEM (*P < .05; *t* test). D, Clustering of colony types in DU145 upon salinomycin treatment (paraclones, red; meroclones, blue; holoclones, green). E, Relative number of each colony type in DU145 and DU145‐DR upon salinomycin treatment. Data represent mean ± SEM (*P < .05; **P < .01; *t* test) [Color figure can be viewed at wileyonlinelibrary.com]

We performed clonogenic assays to evaluate the effects of the stem cell inhibitor salinomycin on the number of colonies. These experiments were conducted in the AR‐negative docetaxel‐sensitive cell lines PC3 and DU145 and their respective docetaxel‐resistant counterparts PC3‐DR and DU145‐DR. Salinomycin treatment significantly decreased the overall colony formation ability of PC3, PC3‐DR, and DU145, but not DU145‐DR (Figure 4A). Detailed analysis and colony classification revealed that salinomycin suppressed the formation of meroclones and holoclones, whereas the amount of paraclones was mostly unchanged by salinomycin (Figure 4B‐E).

## 4 DISCUSSION

Despite the development of novel therapies for advanced PCa, the treatment options for castration‐resistant PCa patients are still limited. Many patients continue to progress after a short period of time and therapy resistance emerges quickly.^18^ Hence, the treatment of advanced PCa remains a major issue and there is an urgent need to identify new therapeutic options to overcome therapy resistance. Common treatments for advanced stages of PCa include androgen deprivation therapy, inhibitors of androgen synthesis and anti‐androgens such as enzalutamide and abiraterone. All these therapies target the AR in highly proliferative cells. Conventional therapies are inefficient in eliminating stem cells, which are AR‐negative^3^ and only show low proliferation and apoptosis rates.^19^ Therefore, it is important to find novel treatment options that eliminate the small population of tumor‐initiating cells that represent the top of the hierarchy in the bulk of PCa cells.

There is still much discussion on how tumor‐initiating PCa cells can be identified and many approaches already exist. In this study, the classification of colony types was performed automatically by the CATCH‐colonies software, which eliminates subjective characterization and leads to reproducible results. The automated classification was confirmed by quantitative real‐time PCR analysis of the stem cell‐related genes Nanog, ALDH1A3, and OCT4 and serial clonogenic assays. Moreover, we demonstrated by immunofluorescence staining that holoclones represent the tumor‐initiating CD24^−^/CD44^high^ population that has previously been described by Al‐Hajj et al.^13^

The therapeutic compound salinomycin is an antibacterial drug that is naturally produced by *Streptomyces albus* and has previously been used as coccidiostat in animals.^20^ The mechanism of action is still not fully elucidated and numerous pathways have been described to be targeted by salinomycin.^12^ Salinomycin has been reported to exert anticancer effects in several tumor entities including PCa.^21^, ^22^, ^23^, ^24^ In detail, it has been shown that gastric cancer stem cells, which were characterized by enhanced Wnt/β‐catenin signaling, are targeted by salinomycin supporting its activity against tumor‐initiating cells.^25^ Moreover, it was demonstrated that salinomycin also decreased the CD24^−^/CD44^+^ stem‐like population in breast cancer cells^26^ and Fuchs et al^27^ observed that salinomycin overcomes apoptosis resistance in human cancer cells. As evidenced in chemoradioresistant nasopharyngeal cancer, salinomycin may inhibit the expression of Nanog protein, in concordance with the results reported in the present study.^28^ It has been reported that salinomycin entered clinical trials^29^; however, no outcome of these studies was reported yet.

Salinomycin significantly decreased the tumor‐initiating CD24^−^/CD44^high^ population and formation of colonies with high tumor‐initiating potential (meroclones/holoclones) in all tested cell lines. This led to a reduction in overall colony formation efficiency in three out of four PCa cells, as it has also been reported by Zhang et al^21^ for RWPE‐1 and PC3 cells. The reduction of colonies was less pronounced in docetaxel‐resistant cells compared to docetaxel‐sensitive cells. A possible explanation for these results could be the high lineage plasticity of prostate basal cells that might be regulated by extrinsic or intrinsic factors, which then may lead to transformation of cells.^30^ Although PC3‐DR and DU145 DR cells do not respond to docetaxel, they show also some phenotypic differences. In this context, it may be mentioned that the basal proliferation rate of PC3‐resistant cells is higher compared to that of their DU145 counterpart.^14^

## 5 CONCLUSIONS

Taken together, we demonstrate that salinomycin specifically targets tumor‐initiating cells in both docetaxel‐sensitive and docetaxel‐resistant cells, which opens new possibilities for the treatment of advanced and castration‐resistant PCa. Targeting the tumor‐initiating population could overcome the problem of the development of therapy resistance and might offer a new treatment strategy in the late stages of PCa.

## CONFLICT OF INTERESTS

The authors declare that there are no conflict of interests.

## Supporting information

Supporting information

Supporting information
