# Supplementary material for: The stem cell inhibitor salinomycin decreases colony formation potential and tumor‐initiating population in docetaxel‐sensitive and docetaxel‐resistant prostate cancer cells
Source: Prostate. 2019 Dec 13;80(3):267–73. doi: 10.1002/pros.23940 (PMC7003856; doi:10.1002/pros.23940)
Supplement: Supplementary file 1 — Supporting information [file PROS-80-267-s001.docx]

**Supplementary Figure Legends**

**Suppl. Figure 1**

**(A)** mRNA levels of stem cell markers Nanog, ALDH1A3 and OCT4 in colony types of DU145 were determined by qPCR. Results represent mean ± SEM from three independent experiments *(*, P*<0.05; *t*-Test)*.* **(B)** Distribution of colonies after subcultivation of individual colony types in PC3 and DU145 (paraclones = red, meroclones = blue, holoclones = green).
