# Supplementary figures and images for: The stem cell inhibitor salinomycin decreases colony formation potential and tumor‐initiating population in docetaxel‐sensitive and docetaxel‐resistant prostate cancer cells
Source: Prostate. 2019 Dec 13;80(3):267–73. doi: 10.1002/pros.23940 (PMC7003856; doi:10.1002/pros.23940)

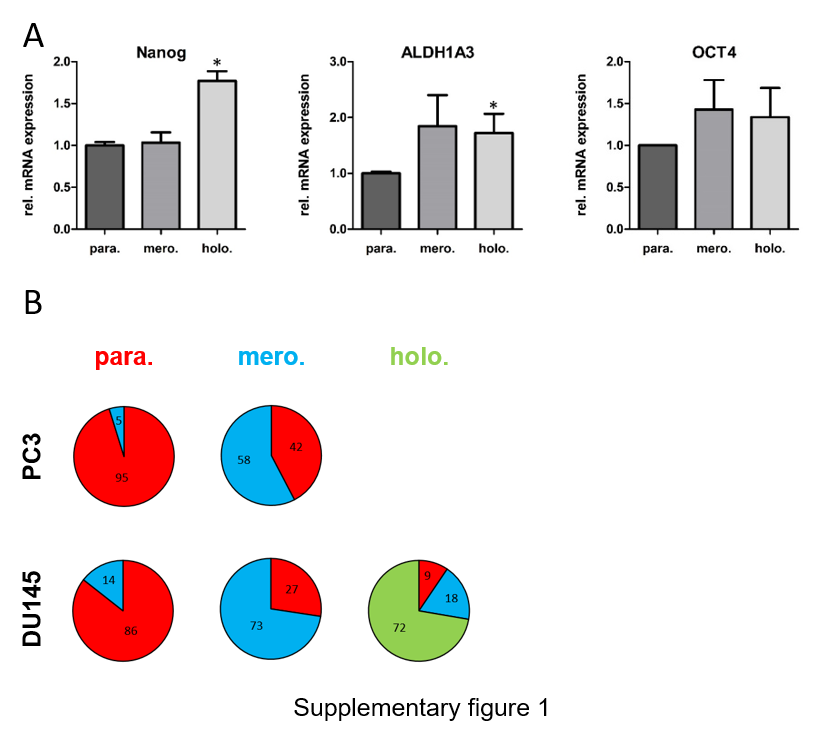

Supplement: Supplementary file 2 — Supporting information [file PROS-80-267-s002.tif]
